# Tumour necrosis factor alpha increases melphalan concentration in tumour tissue after isolated limb perfusion

**DOI:** 10.1054/bjoc.1999.1032

**Published:** 2000-02-01

**Authors:** J H W de Wilt, T L M ten Hagen, G de Boeck, S T van Tiel, E A de Bruijn, A M M Eggermont

**Affiliations:** Department of Surgical Oncology, University Hospital Rotterdam Dijkzigt/Daniel den Hoed Cancer Centre, Groene Hilledijk 301, Rotterdam, EA, 3075, The Netherlands; Laboratory for Organic Chemistry, University of Antwerp, Antwerp, Belgium; Laboratory for Experimental Oncology, University of Leuven, Leuven, Belgium

**Keywords:** TNF, melphalan, tissue concentration, isolated limb perfusion, rats

## Abstract

Several possible mechanisms for the synergistic anti-tumour effects between tumour necrosis factor alpha (TNF-α) and melphalan after isolated limb perfusion (ILP) have been presented. We found a significant sixfold increase in melphalan tumour tissue concentration after ILP when TNF-α was added to the perfusate, which provides a straightforward explanation for the observed synergism between melphalan and TNF-α in ILP. © 2000 Cancer Research Campaign


*British Journal of Cancer* (2000) **82**(5), 1000–1003
© 2000 Cancer Research Campaign
DOI: 10.1054/ bjoc.1999.1032, available online at http://www.idealibrary.com on IDEAL®

Short Communication

### Tumour necrosis factor alpha increases melphalan concentration in tumour tissue after isolated limb perfusion

**JHW de Wilt[1], TLM ten Hagen[1], G de Boeck[2], ST van Tiel[1], EA de Bruijn[3] and AMM Eggermont[1]**

[1]Department of Surgical Oncology, University Hospital Rotterdam Dijkzigt/Daniel den Hoed Cancer Centre, Groene Hilledijk 301, 3075 EA Rotterdam, The Netherlands; [2]Laboratory for Organic Chemistry, University of Antwerp, Antwerp, Belgium; [3]Laboratory for Experimental Oncology, University of Leuven, Leuven, Belgium

**Summary** Several possible mechanisms for the synergistic anti-tumour effects between tumour necrosis factor alpha (TNF-α) and melphalan after isolated limb perfusion (ILP) have been presented. We found a significant sixfold increase in melphalan tumour tissue concentration after ILP when TNF-α was added to the perfusate, which provides a straightforward explanation for the observed synergism between melphalan and TNF-α in ILP. © 2000 Cancer Research Campaign

**Keywords:** TNF; melphalan; tissue concentration; isolated limb perfusion; rats

With isolated limb perfusions (ILP) high drug concentrations can be achieved in the vasculature of a limb with no or negligible leakage into the systemic circulation. With the addition of high dose tumour necrosis factor alpha (TNF-α) to melphalan high response rates were demonstrated in patients with melanoma and irresectable soft tissue sarcomas (Liénard et al, 1992; Eggermont et al, 1996*a*, 1996*b*). Similarly, in rat sarcoma models synergy has been demonstrated between melphalan and TNF-α (Manusama et al, 1996; de Wilt et al, 1999).

The exact mechanisms for synergistic anti-tumour effects between melphalan and TNF-α, however, are not clear. Several possible mechanisms have been suggested such as selective destruction of tumour vasculature, which is accompanied by thrombus formation and haemorrhagic necrosis of the tumour (Shimomura et al, 1988; Renard et al, 1995). This process is accompanied by an inflammatory response that seems to be leucocyte-dependent (Yi and Ulich, 1992; Renard et al, 1994; Manusama et al, 1998). Moreover, TNF-α increases the permeability of tumour vasculature (Folli et al, 1993; Umeno et al, 1994) and has been reported to lower interstitial pressure in the tumour (Kristensen et al, 1996), which could both increase leakage of melphalan in tumour tissue and explain the observed synergy.

To demonstrate this hypothesis we analysed melphalan concentrations in tumour and limb tissue after melphalan isolated limb perfusions with and without the addition of TNF-α.

### MATERIALS AND METHODS

### Chemicals

Melphalan (Alkeran, Wellcome, Beckenham, UK) was diluted in 0.9% sodium chloride and stored at –20°C. Recombinant human TNF-α was provided by Boehringer (Ingelheim, Germany), with specific activity of $5.8 \times 10^7$ U $mg^{-1}$ and endotoxin levels < 1.25 endotoxin units (EU) per mg protein and stored at –80°C. During perfusion 40 μg melphalan with or without 50 μg TNF-α was used.

### Animal tumour model and perfusion setting

Male inbred BN rats, weighing 250–300 g, obtained from Harlan-CPB (Austerlitz, The Netherlands) were used. The perfusion technique was performed as described previously (Manusama et al, 1996). Briefly, small tumour fragments of the non-immunogenic BN-175 soft-tissue sarcoma were implanted in the right hind limb. ILP was performed at a tumour diameter of 13 mm ± 3 mm at least 7 days after implantation. Animals received 50 IU of heparin and the hind limb was kept at a constant temperature of 38–39°C. The femoral artery and vein were cannulated and collaterals were occluded by a groin tourniquet. An oxygenation reservoir was included into the circuit, and melphalan and TNF-α were added as boluses herein. A roller pump recirculated the perfusate at a flow rate of 2.4 ml $min^{-1}$ for 30 min. A washout with 5 ml oxygenated Haemaccel was performed at the end of the perfusion. The committee on Animal Research of the Erasmus University Rotterdam, The Netherlands, approved the experimental protocol.

Tumour growth after perfusion was daily recorded by calliper measurement. Tumour volume was calculated as $0.4(A^2B)$, where A is the minimal tumour diameter and B the diameter perpendicular to A. Tumour volumes were compared 5 days after perfusion.

### Assessment of melphalan concentrations in tissue

Immediately after ILP the perfused tumour and hind limb tissues were excised, homogenized in 2 ml acetonitrile (PRO 200 homogenizer, Pro Scientific, CT, USA), centrifuged at 2500 ***g*** and stored at –80°C. Melphalan was measured by gas chromatography-mass spectrometry (GC-MS), as described previously (De Boeck et al, 1997). P-[Bis(2-chloroethyl)amino]-phenylacetic acid methyl

*Received 23 February 1999*
*Revised 16 June 1999*
*Accepted 21 June 1999*

*Correspondence to:* AMM Eggermont

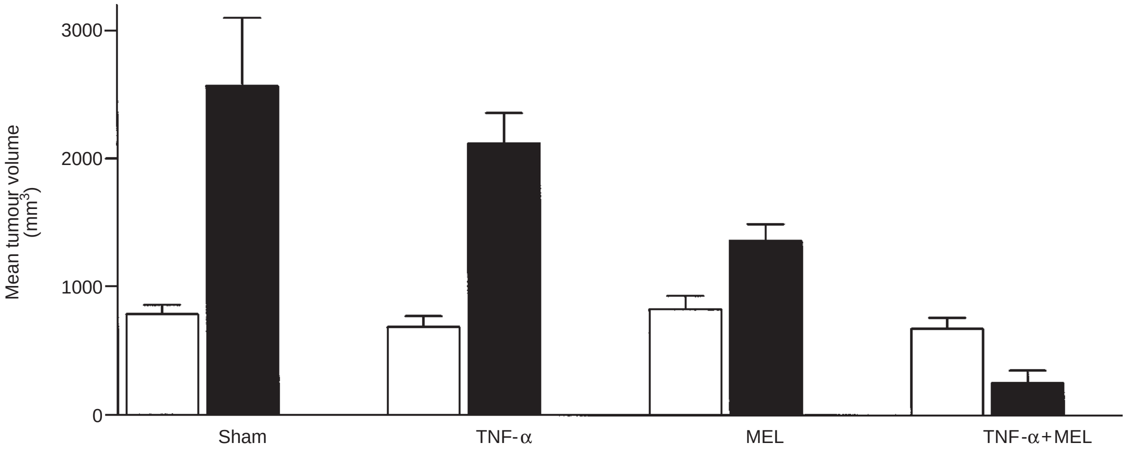

**Figure 1** Mean tumour volumes (± s.e.m.) of BN-175 sarcoma before (□) and 5 days after (■) ILP

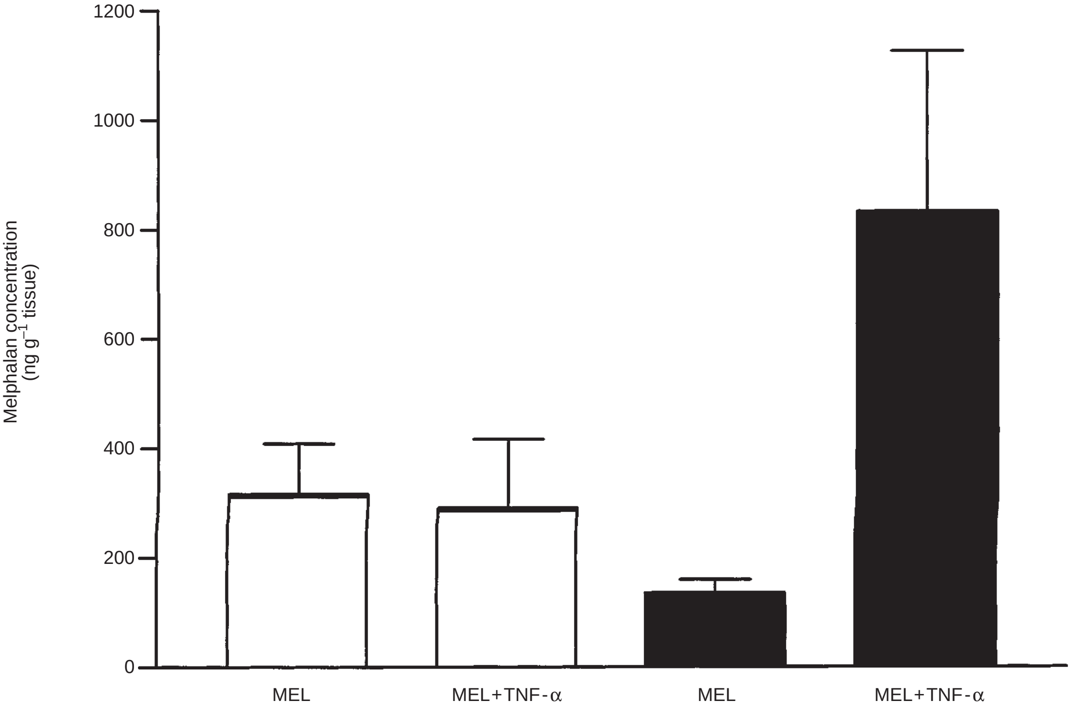

**Figure 2** Melphalan concentration (± s.e.m.) in skin/muscle tissue (□) and tumour tissue (■) immediately excised after ILP with melphalan with or without TNF-α

ester was used as an internal standard. Samples were extracted over trifunctional C18 silica columns. After elution with methanol and evaporation, the compounds were derivatized with trifluoro-acetic anhydride and diazomethane in ether. The stable derivatives were separated on a methyl phenyl siloxane GC capillary column and measured selectively by single ion monitoring GC-MS in the positive EI mode.

### Statistical analysis

Mann–Whitney *U*-test was used to compare tumour volumes in different animal groups and to compare melphalan concentrations in different groups.

### RESULTS

### Tumour response after ILP

Mean tumour volumes were compared to demonstrate the efficacy of ILP with TNF-α and melphalan. Four groups of rats were perfused with sham ($n$ = 10), TNF-α alone ($n$ = 10) melphalan alone ($n$ = 10) and the combination of TNF-α and melphalan ($n$ = 10). A synergistic anti-tumour response was observed with the combination of melphalan and TNF-α as demonstrated before (Manusama et al, 1996; de Wilt et al, 1999) (Figure 1). A significant decrease in mean tumour volume after perfusions with the combination of melphalan and TNF-α was observed ($P < 0.001$), whereas in all other perfusions tumour volume increased.

*© 2000 Cancer Research Campaign* 

### Tissue concentrations of melphalan

Figure 2 demonstrates a sixfold increased melphalan concentration was found in tumour tissue after perfusion with the combination of TNF-α and melphalan ($n$ = 6) in comparison with perfusions with melphalan alone ($n$ = 6) ($P$ = 0.01). TNF-α had no effect on skin and muscle tissue since melphalan concentrations after ILP were comparable with or without the addition of TNF-α.

### DISCUSSION

In the present study we demonstrate an increased accumulation of melphalan in tumour tissue after ILP with the combination of melphalan and TNF-α as compared to melphalan alone. The increased melphalan accumulation could not be demonstrated in skin and muscle tissue, suggesting that TNF-α has no effect on normal tissue. The increased melphalan concentration in tumour tissue correlates very well with the observed tumour response.

The observed responses in this rat soft-tissue sarcoma model are comparable to patients, where TNF-α (Posner et al, 1994) or melphalan alone is not or only marginally active (Klaase et al, 1989). The combination of TNF-α and melphalan, however, results in high response rates (Liénard et al, 1992; Eggermont et al, 1996*a*, 1996*b*). Addition of TNF-α seems crucial in the observed synergy with melphalan and several mechanisms could be responsible for this. A direct effect of TNF-α on the anti-tumour activity of melphalan on BN-175 tumour cells was previously ruled out in vitro (Manusama et al, 1996).

Fajardo et al (1992) previously demonstrated that low-dose TNF-α has a proliferative effect on angiogenesis, whereas higher doses can cause destruction of newly formed blood vessels. It has been demonstrated that this destruction of blood vessels is the result of apoptosis and detachment of angiogenic endothelial cells (Ruegg et al, 1998) which may lead to thrombocyte aggregation, erythrostasis and haemorrhagic necrosis (Shimomura et al, 1988; Renard et al, 1994, 1995; Nooijen et al, 1996).

Whereas previous studies focus on tumour regression resulting from TNF-α-mediated destruction of the vasculature we show that augmented melphalan concentrations in tumour tissue after ILP with TNF-α correlates very well with tumour response and provides an elegant and straightforward explanation for the observed responses. Similarly, drug accumulation in tumour tissue has been shown after systemic pretreatment with TNF-α in mice treated with liposomal doxorubicin (Suzuki et al, 1990; Maruo et al, 1992). An explanation for this phenomenon can be the increased vascular permeability or decreased interstitial pressure that was demonstrated after administration of TNF-α (Folli et al, 1993; Umeno et al, 1994; Kristensen et al, 1996). Alexander et al (1998) demonstrated an increased capillary leakage during isolated hepatic perfusions (IHP) and an increased uptake of I-131 albumin in tumour tissue compared to liver tissue. However, addition of TNF-α did not affect melphalan concentrations in tumour tissue after IHP. Several reasons for this discrepancy are possible such as concentration of TNF-α used, sampling method and duration of perfusion. Another reason can be the difference in tumour vasculature, since colorectal metastases are usually hypovascular and largely necrotic, whereas soft-tissue sarcoma are usually hypervascular.

In conclusion, we hypothesise that increased tumour concentration of melphalan could very well be the main mechanism by which TNF-α enhances the anti tumour response. This finding is not only important for further TNF-α-based limb perfusions using melphalan or other cytostatic agents, but also for other perfusions settings such as isolated liver (Borel Rinkes et al, 1997; Alexander et al, 1998), lung (Pogbreniak et al, 1994) or kidney perfusions (van der Veen et al, 1999).

### ACKNOWLEDGEMENTS

This work was financed in part by a grant from the Dutch Cancer Society. Boehringer Ingelheim GmbH is acknowledged for generously providing TNF-α.

### REFERENCES

Alexander HR, Brown CK, Bartlett DL, Libutti SK, Figg WD, Raje S and Turner E (1998) Augmented capillary leak during isolated hepatic perfusion (IHP) occurs via tumor necrosis factor-independent mechanisms. *Clin Cancer Res* **4**: 2357–2362

De Boeck G, van Cauwenberghe K, Eggermont AMM, van Oosterom AT and de Bruijn EA (1997) Determination of melphalan and hydrolysis products in body fluids by GC-MS. *J High Res Chromat* **20**: 697–700

Borel Rinkes IHM, de Vries MR, Jonker AM, Swaak TJG, Hack CE, Nooijen PTGA, Wiggers T and Eggermont AMM (1997) Isolated hepatic perfusion in the pig with TNF-α with and without melphalan. *Br J Cancer* **75**: 1447–1453

Eggermont AMM, Schraffordt Koops H, Liénard D, Kroon BBR, van Geel AN, Hoekstra HJ and Lejeune FJ (1996*a*) Isolated limb perfusion with high-dose tumor necrosis factor-α in combination with interferon-γ and melphalan for non-resectable extremity soft tissue sarcomas: a multicenter trial. *J Clin Oncol* **14**: 2653–2665

Eggermont AMM, Schraffordt Koops H, Klausner J, Kroon BBR, Schlag PM, Liénard D, van Geel AN, Hoekstra HJ, Meller I, Nieweg OE, Kettelhack C, Ben-Ari G, Pector JC and Lejeune FJ (1996*b*) Isolated limb perfusion with tumor necrosis factor and melphalan for limb salvage in 186 patients with locally advanced soft tissue extremity sarcomas: the cumulative multicenter european experience. *Ann Surg* **224**: 756–765

Fajardo LF, Kwan HH, Kowalski J, Prionas SD and Allison AC (1992) Dual role of tumor necrosis factor-α in angiogenesis. *Am J Pathol* **140**: 539–544

Folli S, Pelegrin A, Chalandon Y, Yao X, Buchegger F, Lienard D, Lejeune FJ and Mach JP (1993) Tumor-necrosis factor can enhance radio-antibody uptake in human colon carcinoma xenografts by increasing vascular permeability. *Int J Cancer* **53**: 829–836

Klaase JM, Kroon BBR, Benckhuijsen C, van Geel AN, Albus-Lutter CE and Wieberdink J (1989) Results of regional isolation perfusion with cytostatics in patients with soft tissue tumors of the extremities. *Cancer* **64**: 616–621

Kristensen CA, Nozue M, Boucher Y and Jain RK (1996) Reduction of interstitial fluid pressure after TNF-alpha treatment of three human melanoma xenografts. *Br J Cancer* **74**: 533–536

Liénard D, Ewalenko P, Delmotte JJ, Renard N and Lejeune FJ (1992) High-dose recombinant tumor necrosis factor alpha in combination with interferon gamma and melphalan in isolation perfusion of the limbs for melanoma and sarcoma. *J Clin Oncol* **10**: 52–60

Manusama ER, Nooijen PTGA, Stavast J, Durante NMC, Marquet RL and Eggermont AMM (1996) Synergistic antitumour effect of recombinant human tumour necrosis factor α with melphalan in isolated limb perfusion in the rat. *Br J Surg* **83**: 551–555

Manusama ER, Nooijen PTGA, Stavast J, de Wilt JHW, Marquet RL and Eggermont AMM (1998) Assessment of the role of neutrophils on the antitumor effect of TNF-α in an in vivo isolated limb perfusion model in sarcoma-bearing brown Norway rats. *J Surg Res* **78**: 169–175

Maruo Y, Konno H and Baba S (1992) Therapeutic effects of liposomal adriamycin in combination with tumor necrosis factor-α. *J Surg Oncol* **49**: 20–24

Nooijen PTGA, Manusama ER, Eggermont AMM, Schalkwijk L, Stavast J, Marquet RL, de Waal RMW and Ruiter DJ (1996) Synergistic antitumour effects of TNF-α and melphalan in an isolated limb perfusion model of rat sarcoma: a histopathologic, immunohistochemical and electron microscopic study. *Br J Cancer* **74**: 1908–1915

Pogrebniak HW, Witt CJ, Terrill R, Kranda K, Travis WD, Rosenberg SA and Pass HI (1994) Isolated lung perfusion with tumor necrosis factor: a swine model in preparation of human trials. *Ann Thorac Surg* **57**: 1477–1483

Posner M, Liénard D, Lejeune FJ, Rosenfelder D and Kirkwood J (1994) Hyperthermic isolated limb perfusion (HILP) with tumor necrosis factor alpha

 *© 2000 Cancer Research Campaign*

(TNF) alone for metastatic in-transit melanoma. *Proc Annu Meet Am Soc Clin Oncol* **13**: A1351

Renard N, Liénard D, Lespagnard L, Eggermont AMM, Heimann R and Lejeune FJ (1994) Early endothelium activation and polymorphonuclear cell invasion precede specific necrosis of human melanoma and sarcoma treated by intravascular high-dose tumour necrosis factor alpha (rTNFα). *Int J Cancer* **57**: 656–663

Renard N, Nooijen PTGA, Schalkwijk L, de Waal RMW, Eggermont AMM, Liénard D, Kroon BBR, Lejeune FJ and Ruiter DJ (1995) VWF release and platelet aggregation in human melanoma after perfusion with TNFα. *J Pathol* **176**: 279–287

Ruegg C, Yilmaz A, Bieler G, Bamat J, Chaubert P and Lejeune FJ (1998) Evidence for the involvement of endothelial cell integrin alphaVbeta3 in the disruption of the tumor vasculature induced by TNF and IFN-gamma. *Nat Med* **4**: 408–414

Shimomura K, Manda T, Mukumoto S, Kobayashi K, Nakano K and Mori J (1988) Recombinant human tumor necrosis factor-α: thrombus formation is a cause of anti-tumor activity. *Int J Cancer* **41**: 243–247

Suzuki S, Ohta S, Takashio K, Nitanai H and Hashimoto Y (1990) Augmentation for intratumoral accumulation and anti-tumor activity of liposome-encapsulated adriamycin by tumor necrosis factor-alpha in mice. *Int J Cancer* **15**: 1095–1100

Umeno H, Watanabe N, Yamauchi N, Tsuji N, Okamoto T and Niitsu (1994) Enhancement of blood stasis and vascular permeability in Meth-A tumors by administration of hyperthermia in combination with tumor necrosis factor. *Jpn J Cancer Res* **85**: 325–330

Van der Veen AH, Durante NMC, Breurs J, Nooijen PTGA, Marquet RL and Eggermont AMM (1999) In vivo isolated kidney perfusion with TNF-α in tumour bearing rats. *Br J Cancer* **79**: 433–439

de Wilt JHW, Manusama ER, van Tiel ST, van IJken MGA, ten Hagen TLM and Eggermont AMM (1999) Prerequisites for effective isolated limb perfusion using tumour necrosis factor alpha and melphalan in rats. *Br J Cancer* **80**: 161–166

Yi E and Ulich T (1992) Endotoxin, interleukin-1, and tumor necrosis factor cause neutrophil-dependent microvascular leakage in postcapillary venules. *Am J Pathol* **140**: 659–663

*© 2000 Cancer Research Campaign*

